# Associations between Autoimmunity and Depression: Serum IL-6 and IL-17 Have Directly Impact on the HAMD Scores in Patients with First-Episode Depressive Disorder

**DOI:** 10.1155/2022/6724881

**Published:** 2022-05-16

**Authors:** Lijuan Mao, Xiajin Ren, Xin Wang, Feng Tian

**Affiliations:** Psychiatry Department, Second Hospital of Shanxi Medical University, No. 382, Wuyi Road, Taiyuan, 030001 Shanxi Province, China

## Abstract

**Objective:**

The study is aimed at evaluating the immune-activation state before and after treatment in patients with first-episode depressive disorder (FDD) with evaluating the ILs and CRP levels and further clarifying the association between autoimmunity and the etiology and pathogenesis of FDD.

**Methods:**

We designed a case-control study. FDD patients and healthy subjects were enrolled in the FDD group and control group. Serum IL-6, IL-17, and CRP were measured before and after selective serotonin reuptake inhibitor (SSRI) therapy, as well as Hamilton rating scale for depression (HAMD) and life event scale (LES) scores. The correlations between IL-6 and IL-17 and HAMD and LES scores were analysed, and multiple linear regression analysis was performed for HAMD score.

**Results:**

40 FDD patients and 40 healthy subjects were included in the FDD and control group from October 2009 to September 2012. Before treatment, the IL-6 (28.99 ± 5.51, *P* < 0.001) and IL-17 (41.15 ± 4.80, *P* < 0.001) in the FDD group were significantly higher than the control group (16.84 ± 3.78 and 21.68 ± 3.72, respectively). The C-reactive protein (CRP) level in two groups was comparable (*P* = 0.879). After treatment, the IL-6 (18.69 ± 5.07, *P* < 0.001) and IL-17 (30.67 ± 3.47, *P* < 0.001) levels and HAMD scores (6.73 ± 4.15) in the FDD group were significantly decreased than before treatment (*P* < 0.001, respectively). CRP level was slightly increased after treatment without statistically significant (*P* = 0.239). The HAMD score correlated with IL-6 (*r* = 0.638, *P* < 0.001) and IL-17 (*r* = 0.927, *P* < 0.001); the total LES and negative LES also correlated with IL-6 (*r* = 0.226, *P* < 0.05) (*r* = 0.366, *P* <0.001) and IL-17 (*r* = 0.348, *P* < 0.001) (*r* = 0.493, *P* < 0.001). Multiple linear regression analysis showed that both of the IL-6 and IL-17 had direct impact on HAMD score.

**Conclusion:**

The autoimmunity status was overactivated in FDD patients, and serum IL-6 and IL-17 levels had direct impact on the HAMD score. Patients who experienced more negative life events had higher activation level of autoimmunity status and HAMD scores, and serum IL-6 and IL-17 levels can be decreased by SSRI treatment.

## 1. Introduction

Depressive disorder is a common mental disorder. The main symptoms are low mood, loss of interest, low self-evaluation, and decreased volition and behavior. In severe cases, it is manifested as pessimism and suicide [1]. Its lifetime prevalence exceeds 15% [2]. Studies have shown that depressive disorder is related to genetic, biochemical, and psychosocial factors [3–5].

Cytokine-mediated immune dysfunction has become one of the hot spots in the pathogenesis of depression [5]. Increased release of IL-6 in major depressive disorder (MDD) has been found to be a factor associated with MDD prognosis and therapeutic response and may affect a wide range of depressive symptomatology [6]. IL-17 is the founding member of a novel family of inflammatory cytokines, which is an early promoter of T-cell-induced inflammatory response and can amplify the inflammatory response by promoting the release of proinflammatory cytokines [7]. Some studies have found that in elderly patients with coronary heart disease complicated with depression, the increase of serum IL-17 concentration is more obvious with the aggravation of depression degree [8, 9].

At present, the etiology and pathogenesis of depression are still remained unclear; although abnormal immune dysfunction and autoimmunity has been suspected as one of the reason, the association between IL-6 and IL-17 and the etiology as well as pathogenesis of depressive disorder is unclear. Herein, in this study, we aimed to determine how the serum IL-6 and IL-17 changes in patients with first-episode depressive disorder (FDD) before and after treatment and finally determine the association between autoimmunity and FDD.

## 2. Materials and Methods

### 2.1. Subjects

We designed a case-control study. From October 2009 to September 2012, the FDD patients who were hospitalized and outpatient in the Mental Health Department of the First and Second Hospital of Shanxi Medical University were enrolled in the FDD group. FDD was diagnosed according to the diagnostic criteria for depressive disorder in the Diagnostic and Statistical Manual of Mental Disorders- (DSM-) 5 [10]. Inclusion criteria included the following: (1) Han nationality, 18-60 years old; (2) the Hamilton rating scale for depression (HAMD) ≥ 18 [11]; and (3) has signed the consent form and voluntarily participated in the study. The exclusion criteria are as follows: (1) patients who suffered from schizophrenia or other psychotic disorders; (2) patients with alcohol or drug abuse; (3) female patients in pregnancy or lactation; (4) patients whose laboratory tests (blood routine, liver function, kidney function) or electrocardiograph were obviously abnormal; (5) patients with endocrine diseases, such as hypothyroidism; (6) patients who had taken antidepressants (e.g., tricyclic monoamine oxidase inhibitors and selective 5-hydroxytryptamine receptor blockers) or mood stabilizers (e.g., lithium, valproate, and carbamazepine) within a year; (7) patients who had taken immunomodulatory agents or hormone preparations within 6 months or any psychotropic medication or nonsteroidal anti-inflammatory drugs (NSAIDs) within 2 weeks; (8) patients with a history of acute infection, trauma, inflammation, fever, or allergy within 2 weeks; and (9) patients who had received electroconvulsive therapy or sleep deprivation therapy for the past 2 weeks. Healthy subjects were enrolled in the control group after matching sex and age with the FDD group. The other basic characteristics included the educational and marital status. All patients in the FDD group received 6 weeks of selective serotonin reuptake inhibitors (SSRIs).

The protocols and procedures for the protection of human subjects were approved by the Ethics Committee (IRB ethical approval: KY-2009-OB02), and all of the methods were carried out in accordance with approved guidelines.

### 2.2. Laboratory Tests

Serum IL-6, IL-17, and CRP levels were tested in the FDD group and control group. Blood samples were collected on the morning of the day before treatment and the 42th day after treatment in the FDD group. IL-6, IL-17, and CRP were detected by enzyme-linked immunosorbent assay (ELISA) with human IL-6, IL-17, and CRP ELISA kits (Boster Biological Technology Co. Ltd., Wuhan, China).

### 2.3. Life Event Scale (LES)

The LES were assessed in the FDD group before the treatment, as well as the control group. LES is a self-rating scale [12], which is the only life event scale in China that separately evaluates the impact of positive and negative life events. LES scale included three aspects: family life, work and study, and social interaction and others [12]. The patients filled it out according to their own actual feelings, instead of judging by common sense or ethical concepts that what they had experienced is bad or good. Finally, the family, work-study, and social event stimulus, as well as the positive, negative, and total life event stimulus, were calculated. The stimulus of each event = the influence degree × the duration × the occurrence number. The stimulus amount of positive/negative life events = sum of all positive/negative stimuli. The total stimulus = positive stimulus of life events + negative stimulus of life events.

### 2.4. HAMD Score

HAMD score is used to define the clinical severity of the depression and the varied. The HAMD score was performed before and after the SSRI treatment (6 weeks) in the FDD group.

### 2.5. Statistical Analysis

Continuous data were expressed as the mean ± standard deviation (SD) and compared by independent sample *t*-tests and Levene variance homogeneity tests. Count data were expressed as number or percentage (%) and compared by a Chi-squared test or Fisher's exact test. The significance level was set at 0.05 (two-tailed).

Spearman correlation analysis was performed between parameters, and multiple linear regression analysis was performed to determine the independent factors of HAMD scores. All of the statistical analyses were performed using IBM SPSS 20.0.0 (SPSS Inc., 2009, Chicago, IL, USA).

## 3. Results

### 3.1. Basic Characteristics

Finally, 40 FDD patients were included in the FDD group, and 40 healthy subjects were included in the control group after matching. All of the subjects had completed the study without missing. The age, sex, educational, and marital status between the two groups did not have significant difference (Table 1). The total LES and negative LES were much higher in the FDD group than the control group (Table 1).

### 3.2. The IL-6, IL-17, and HAMD Scores before and after SSRI Treatment

Before treatment, the IL-6 and IL-17 levels in the FDD group were significantly higher than those in the control group (Table 2). After treatment, the IL-6 and IL-17 levels in the FDD group were decreased than before (Table 2); however, the IL-17 in the FDD group was still higher than the control, while the IL-6 level in the FDD group was comparable with the control group (Table 2). The CRP had no difference in the FDD and control groups (Table 2). The HAMD scores were significantly decreased after the treatment than before (Table 2).

### 3.3. Correlation Analysis of IL-6 and IL-17 and HAMD and LES Scores

The total HAMD score had a medium strength correlation with IL-6 and a strong correlation with IL-17 (Table 3). The total LES had mild correlation with IL-6 and IL-17, and the negative LES had a mild correlation with IL-6 and a medium strength correlation with IL-17 (Table 3).

### 3.4. Multiple Linear Regression Analysis for the HAMD

Multiple linear regression analysis showed that both of the IL-6 and IL-17 were independent factors affecting HAMD score (Table 4). CRP had no direct impact on the HAMD score (Table 4).

## 4. Discussion

In physiological conditions, the immune cells are in a relatively static state. When the body is subjected to external stimuli (such as stress, bacteria, and viruses) or internal influence, the immune cells are abnormally activated, and the secretion of inflammatory cytokines increases. The body cannot maintain its homeostasis of the internal environment through its own regulation; thus, a series of immune responses appear. Current studies tend to believe that patients with depression are in the immune-activated state [13, 14]. A previous study suggested that the pathogenesis of depression not only has the change of neurotransmitter function but also has a close relationship with the change of immune function [15]. A meta-analysis of RCTs of NSAIDs, administered as sole treatment or as adjunct to antidepressants, indicates that they are more effective than placebo in treating depression [16].

IL-6 and IL-17 are proinflammatory cytokines that play an important role in the early stage of the immune response and are important cytokines involved in the neuro-immune-endocrine system network [17]. IL-6 is a pleiotropic cytokine involved in the onset and resolution of inflammation and responses to infection, tissue remodelling, and cancer. It is produced by fibroblasts, endothelial cells, macrophages, and lymphocytes [18]. IL-17 is produced by helper T (Th) cells that are stimulated by IL-1*β*, IL-6, and signal transducer and activator of transcription 3 (STAT3) derived from phagocytes such as macrophages and from tissue cells [18, 19], and through the positive feedback loop to enhance the expression and/or activation of IL-6, IL-17, and STAT3 [18]. Furthermore, the differentiation of Th17 cells can be completely blocked by IL-6 neutralizing antibody, and IL-6 neutralizing antibody can also inhibit the occurrence and development of autoimmune diseases mediated by Th17 cells [20]. So far, there are many studies on the expression of serum IL-6 of depressive disorder, but its integration into routine clinical care has not yet been fully elucidated [6, 21, 22].

The results of this study showed that the levels of serum IL-6 and IL-17 in patients with FDD before treatment were higher than those after SSRI antidepressants treatment and the control group. After treatment, the level of IL-17 was higher than that of the control group, and the level of IL-6 after treatment was of no significant difference than that of the control group. These further confirmed that patients with depressive disorder had abnormal cytokine levels and were in a state of immune activation. SSRIs have been shown to improve depression and normalize cellular immune systems. After SSRI antidepressant treatment, the total HAMD score decreased, and the symptoms improved significantly. The total HAMD score in patients with depressive disorder before treatment was positively correlated with serum IL-17, but not with IL-6. The difference of HAMD score before and after treatment was positively correlated with the variation of IL-17 concentration. The more IL-17 concentration decreased, the more HAMD score decreased, and the more obvious symptom improvement.

The subjects of this study were FDD patients who did not take medication, suggesting that changes in serum IL-6 and IL-17 levels have been manifested in the early stages of depressive disorder, and IL-17 is closely related to the severity of depressive disorder. It can be speculated that IL-17 may be used as a biological indicator for early diagnosis of patients with depression, as well as one of the indicators for symptom remission of patients with depression.

Accumulating evidence indicate a role for the autoimmunity in the aetiology of depression [23]. IL-17 has received much attention for its proinflammatory role in autoimmune disease [24]. A variety of drugs targeting the IL-17 pathway have been proven to be effective in treating psoriasis [25]. Qiu et al. [26] found that serum glucocorticoid receptors in patients with depression were lower than those in the control group, especially in patients with severe depression, which significantly reduced the number and function of regulatory T cells, leading to abnormal proliferation and increased activity of their own response cells. Harbuz et al. [27] also showed that glucocorticoid resistance plays an important role in autoimmune injury. Combined with the abnormal expression of IL-17 in patients with FDD in this study, it is suggested that patients with depression may have the tendency of autoimmune dysfunction, and further studies on related antibodies in patients with depression are needed to confirm this.

In MDD, there is evidence of inflammatory activation and increased production of proinflammatory cytokines, leading to the synthesis of proteins in the acute phase, which are sensitive markers of systemic inflammation, such as CRP [28]. However, previous studies have proved that there was no significant difference in CRP concentrations between the MDD patients and healthy controls [28–30], which is consistent with our findings.

The average intensity of negative life events experienced by patients with depression disorder is higher than that of normal people, suggesting that life events may be directly related to the occurrence of depression disorder [31]. Depression is associated with less social support, which can buffer negative stimuli and affect the way patients respond to stimuli, thus promoting individuals to better adapt to the surrounding environment [32]. One of the important causes of depression is psychological stress, which can lead to immune activation [33]. Psychological stress can affect the secretion of brain and peripheral cytokines and participate in the pathogenesis of depression [33]. The results of this study showed that the level of serum IL-6 and IL-17 in patients with depression before treatment was positively correlated with the amount of negative life event stimulation experienced, but not with the amount of positive life event stimulation experienced. As social support increased, the HAMD score decreased. Qiu et al. [34] found a significant positive correlation between serum IL-6 level and life event intensity in female patients with depression, which is consistent with the results of this study. It can be seen that experiencing negative life events is very important for the occurrence of depression disorder. Social support can improve patients' depressive symptoms to a certain extent. Patients should be encouraged to contact with others more, so as to get more social support, which has a certain effect on the remission of the condition.

This prospective study had some limitations. First, the sample size enrolled in this study was relatively small; hence, the conclusion needed further verification. Second, this study mainly provides the observation results of a clinical phenomenon, and further research is needed to explore the mechanism. Third, despite the low incidence of other diseases associated with immune activation, the failure to completely exclude these diseases before enrollment may also have affected the results of this study to a certain extent.

## 5. Conclusion

The autoimmunity status was overactivated in FDD patients, and serum IL-6 and IL-17 levels had direct impact on the HAMD score. Patients who experienced more negative life events had higher activation level of autoimmunity status and HAMD scores, and serum IL-6 and IL-17 levels can be decreased by SSRI treatment.

## Figures and Tables

**Table 1:** Basic characteristics of the control and first-episode depressive disorder (FDD) group.

Basic characteristics	Control group (*n* = 40)	FDD group (*n* = 40)	t/*χ*^2^	*P* value
Age (years)	34.85 ± 11.62	36.55 ± 11.97	−0.645	0.521
Sex (n,%)			0.487	0.485
Male	16 (40.0)	13 (32.5)		
Female	24 (60.0)	27 (67.5)		
Education (n,%)			0.814	0.797
Junior high school and below	5 (12.5)	4 (10.0)		
Senior high school	19 (47.5)	16 (40.0)		
Bachelor degree and above	16 (40.0)	20 (50.0)		
Marital status (n,%)			1.008	0.604
Never married	10 (25.0)	12 (30.0)		
Married	27 (67.5)	23 (57.5)		
Previously married	3 (7.5)	5 (12.5)		
Total LES	16.40 ± 22.74	38.80 ± 44.86	−2.817	0.007^∗∗^
Positive LES	8.43 ± 15.71	4.35 ± 7.86	1.467	0.148
Negative LES	7.89 ± 14.00	34.45 ± 39.49	−3.977	<0.001^∗∗^

Note: FDD, first-episode depressive disorder, LES, Life event scale; Sex, education, and marital status were analyzed by Fisher exact test; ^∗∗^*P* < 0.01.

**Table 2:** Comparisons in control group and FDD group before and after the treatment.

Index	Control group (*n* = 40)	FDD group (*n* = 40)		*P* value t value		
		Before treatment (*n* = 40)	After treatment (*n* = 40)	Control vs before	Control vs after	Before vs after
IL-6 (pg/mL)	16.84 ± 3.78	28.99 ± 5.51	18.69 ± 5.07	<0.001^∗∗^ t = −11.488	0.068 t = −1.850	<0.001^∗∗^ t = 11.083
IL-17 (pg/mL)	21.68 ± 3.72	41.15 ± 4.80	30.67 ± 3.47	<0.001^∗∗^ t = −20.278	<0.001^∗∗^ t = −11.177	<0.001^∗∗^ t = 12.272
CPR (mg/L)	2.83 ± 2.37	2.92 ± 2.51	3.58 ± 2.51	0.879 t = −0.153	0.175 t = −1.370	0.239 t = −1.186
HAMD scores	—	20.78 ± 3.10	6.73 ± 4.15	—	—	<0.001^∗∗^ t = 20.597

Note: IL, interleukin; FDD, first-episode depressive disorder; HAMD, Hamilton rating scale for depression; ^∗∗^*P* < 0.01.

**Table 3:** Correlations between IL-6, IL-17 and LES and HAMD scores.

Scales	IL-6 (*n* = 80)	IL-17 (*n* = 80)
Total HAMD score	*r* = 0.638^∗∗^	*r* = 0.927^∗∗^
Total LES score	*r* = 0.226^∗^	*r* = 0.348^∗∗^
Positive LES score	*r* = −0.074	*r* = −0.064
Negative LES score	*r* = 0.366^∗∗^	*r* = 0.493^∗∗^

Note: IL, interleukin; FDD, first-episode depressive disorder; HAMD, Hamilton rating scale for depression; LES, Life event scale; ^∗∗^*P* < 0.01. ^∗^*P* < 0.05.

**Table 4:** Multiple linear regression analysis for the HAMD.

Parameters	B	SEM	*P*-value	VIF
IL-6	0.097	0.033	0.004^∗∗^	1.764
IL-17	1.023	0.066	<0.001^∗∗^	1.760
CRP	−0.060	0.129	0.643	1.039

Note: R^2^ = 0.878. SEM = standard error of mean; VIF = variance inflation factor; IL, interleukin; HAMD, Hamilton rating scale for depression; ^∗∗^*P* < 0.01.

## Data Availability

Data are available on request.
